# Hybrid 12-Month Exoskeleton Training with Percutaneous Epidural Stimulation After Spinal Cord Injury

**DOI:** 10.3390/life16010077

**Published:** 2026-01-04

**Authors:** Jakob N. Deitrich, Muhammad Uzair Rehman, Robert Trainer, David X. Cifu, Ashraf S. Gorgey

**Affiliations:** 1 Spinal Cord Injury and Disorders Center, Richmond VA Medical Center, Richmond, VA 23249, USArehmanu@vcu.edu (M.U.R.); 2 Department of Biomedical Engineering, Virginia Commonwealth University, Richmond, VA 23284, USA; 3 Physical Medicine and Rehabilitation, Richmond VA Medical Center, Richmond, VA 23249, USA; robert.trainer@va.gov; 4 Department of Physical Medicine and Rehabilitation, Virginia Commonwealth University, Richmond, VA 23284, USA

**Keywords:** exoskeleton training, percutaneous spinal cord epidural stimulation (SCES), spinal cord injury (SCI), permanent implantation, spinal mapping

## Abstract

**Background**: Exoskeleton-assisted walking (EAW) is an effective method of rehabilitation that improves ambulation as well as independence in individuals with chronic spinal cord injury (SCI). Percutaneous spinal cord epidural stimulation (SCES) is another strategy that has shown promising results for promoting locomotor recovery in persons with SCI. We hypothesized that the hybrid application of SCES with EAW for 12 months would further enhance motor performance after SCI. **Methods**: Four males with chronic motor complete SCI underwent permanent implantation with percutaneous SCES. Participants were assigned to either 12 months of EAW + SCES or EAW without SCES [three times weekly] for the first 6 months, followed by an additional 6 months of EAW + SCES + resistance training (RT) or EAW+ delayed SCES + no RT [3–5 times per week]. Weekly exoskeleton data were captured and averaged for every 2–3 sessions using the Ekso Pulse platform to measure exoskeleton performance parameters. Exoskeleton assistance modes started with fixed assistance for the first six months of the trial and were followed by another six months of adaptive assistance. **Results**: The four participants completed 41 ± 7 weeks of the designated 48 weeks (85%). The up time across the entire study was 61 ± 6 min, and the EAW time was 46 ± 3 min. The overall number of steps, distance, and speed were 1315 ± 291, 426 ± 113 m, and 0.16 ± 0.04 m/s, respectively. Steps per minute, EAW distance, and speed were higher during sessions with the adaptive-assistance mode compared to the fixed mode. However, this was accompanied by a decrease in the level of assistance or percentage of minimum assistance provided by the exoskeleton. **Conclusions**: Twelve months of hybrid EAW with SCES resulted in an increase in steps per minute, distance, and EAW speed during adaptive mode in persons with SCI. The findings may serve as the basis for future studies to consider hybrid integration of exoskeletons with neuromodulation techniques to further enhance rehabilitation potential after SCI.

## 1. Introduction

Robotic exoskeleton walking has been recognized as an effective rehabilitation intervention for gait re-education after spinal cord injury (SCI) [1,2,3]. Several reports demonstrated the efficacy of exoskeletal-assisted walking (EAW) in improving ambulation, increasing the level of physical activity and quality of life, as well as decreasing the likelihood of developing secondary comorbidities after SCI [2,3,4,5]. EAW maintains high therapist–patient interaction by customizing training based on the needs of each participant while decreasing the demands on staff [1,5,6]. Furthermore, robotic exoskeletons are recognized for their low metabolic costs during walking, providing an opportunity for unlimited repetitions of task-specific movements and the capability for repeated activation of the neuromuscular system by selectively inducing a fixed proprioceptive pattern [7]. This pattern is likely to provide a flux of sensory information to the dormant lumbosacral segments and enhance gait re-education after SCI [8,9,10]. However, the effectiveness of this approach in restoring motor control is still questionable, especially in persons with complete SCI. Controversial findings have been demonstrated following EAW training in persons with incomplete SCI compared to complete SCI [2,11]. A recent randomized controlled trial demonstrated that EAW for 60 days did not improve gait parameters or postural control after incomplete SCI [11].

To address the aforementioned limitations, several scientific attempts have been geared towards applications of hybrid rehabilitation technologies [8,12,13]. Hybrid use of exoskeletons with electrical stimulation has been successfully shown to actively engage paralyzed muscles to control their movement after SCI [14,15]. The process of synchronizing exoskeleton walking with electrical stimulation technology resulted in less muscle fatigue, increased the number of repetitions of specific tasks, and enhanced the mechanical efficiency of the movement [14,15]. This has led researchers to integrate a neuromodulation approach in either an open or closed loop format to enhance gait re-education during EAW, allowing participants with several neurological disorders to actively assist with robotic movements during overground ambulation [8,9,13]. This human–EAW interface has resulted in several proof-of-concept studies that presented the possibility for paralyzed individuals to actively reduce robotic assistance and generate steps despite years of injury [12,16,17]. Additionally, previous trials demonstrated that neuromodulation interventions augmented task-specific training or robotic stepping in a frequency-dependent manner; this may have helped differentiate between tonic stimulation to achieve standing and spontaneous rhythmic stimulation to achieve stepping [18,19]. For example, Gad et al. demonstrated that non-invasive trans-spinal stimulation (TSS) resulted in decreased minimum adaptive assistance required by EAW in a blind person with SCI [16]. Sutor et al. demonstrated that the percentage of robotic-assisted steps decreased when TSS activated the lumbosacral segments at sub-motor thresholds in three persons with motor complete SCI [17]. The authors further showed increased EMG activity of the hip muscles during the 10 m EAW test [17]. Another study demonstrated that spinal cord epidural stimulation (SCES) paired with EAW for 12 weeks resulted in an increase in the number of active steps by decreasing the fixed assistance to 35% during EAW in a person with SCI [12]. However, the case report was limited to 12 weeks with sessions occurring only twice weekly and did not exercise all the assisted spectrums (i.e., fixed versus adaptive assistance) provided by robotic exoskeletons [12].

The REST-SCI trial was conducted to examine the effects of 12 months of percutaneous spinal cord epidural stimulation (SCES) with EAW on motor performance and overground ambulation after SCI [13,20]. The initial report demonstrated the successful hybrid engagement between EAW and SCES in achieving sit-to-stand activity [20]. EAW provided active assistance at the level of hips and knees, while a participant with complete SCI could achieve standing using the squat mode only with SCES on [20]. Another successful feature is the capability of SCES rhythmic configurations to enhance EAW performance, as measured by steps per minute, step length, and minimum assistance in two persons with complete SCI [20]. However, limited evidence is available about a long-term synergistic interaction between EAW and SCES, especially with the possibility of providing either fixed or adaptive assistance. Therefore, the aim of the study was to descriptively present the findings from 12 months of EAW with SCES to enhance robotic performance in persons with complete or motor complete SCI. For the first 6 months, the EAW assistance level was set primarily at fixed assistance. EAW assistance was then set only in adaptive mode during the remaining six months to ensure the capture of variable gait parameters based on individual performance across the gait cycle.

## 2. Methods

### 2.1. Subjects

The randomized clinical trial was approved by the Richmond Veterans Affairs Medical Center, and four men with clinically motor complete SCI (Table 1) participated in the study [13,20]. All methods abided by the relevant guidelines and regulations, and the trial was registered with the registration I.D. # NCT04782947 at clinicaltrials.gov. Written and verbal consent was obtained from each participant for all study procedures. Participants received detailed physical and medical screening from a SCI-certified physician according to the International Standards for Neurological Classification of Spinal Cord Injury (ISNCSCI), including a modified Ashworth scale assessment to ensure inclusion criteria are met. The study timeline has been published previously, detailing all aspects of the trial and inclusion–exclusion criteria [13]. Table 2 provides a detailed timeline for each participant across the entire trial.

Briefly, all participants were 18–60 years old, male or female, with traumatic motor complete SCI [AIS A and B] and level of injury below C5, as determined by the International Standards for Neurological Classification of SCI (ISNCSCI) exam. A written clearance by the medical provider was required to ensure safety prior to enrollment in the program.

Participants with any of the following pre-existing medical conditions were excluded from the current trial: (1) diagnosis of neurological injury other than SCI, including cauda equina or distal conus injuries resulting in limb or sacral areflexia; (2) unhealed fracture in either lower or upper extremities; (3) severe scoliosis, hip–knee range of motion or flexion knee contractures preventing positioning in an exoskeleton, or plantarflexion contracture greater than 20 degrees; (4) untreated or uncontrolled hypertension, defined as high resting blood pressure greater than 140/90 mmHg and severe orthostatic hypotension (drop greater than 20 mmHg compared to resting supine blood pressure) or incapable of maintaining a sitting or EAW standing posture; (5) other medical conditions including cardiovascular disease, uncontrolled type II DM, and those on insulin, or with symptomatic urinary tract infection; (6) taking anti-coagulants or anti-platelet agents, including aspirin, if unable to be off this medication for medical reasons; (7) implanted pacemakers and/or implanted defibrillator devices; (8) DXA T-scores or BMD less than −2.5, −3.5 SD, and 0.6 g/cm^2^, respectively, for total body, dual hips, and knees; (9) participants with severe spasticity or limited ROM, as measured by a modified Ashworth scale; (10) pressure ulcer of the trunk, pelvic area, or lower extremities of grade 3 or more; (11) psychopathology documentation in the medical record or history that may conflict with study objectives; (12) allergic reaction to the antibiotics minocycline and rifampin; (13) pregnant women and women who may become pregnant during the course of the study; (14) any condition that, in the judgment of the principal investigator or medical provider, precludes safe participation in the study and/or increases the risk of infection.

### 2.2. Study Timeline

The study followed participants for approximately 12 months with the intended goal of examining the impact of implanted percutaneous SCES paired with EAW as well as resistance training (RT) on motor control in individuals with chronic SCI. Participants underwent spinal mapping following implantation during the trial [13,20]. Mapping was performed to determine optimal SCES configurations and stimulation parameters for each participant [21,22]. Three participants [participants 0881, 0082, and 0884] underwent six months of EAW + SCES followed by six months of EAW + SCES + RT. The remaining participant [0883] underwent six months of only EAW, then was implanted with percutaneous SCES and proceeded for an additional 6 months of EAW + SCES + no RT. The assignments were based on pre-determined randomization of our participants into two interventional groups. The timeline for each group assignment was recently published [see ref. [13]; Figure 2].

The study rationale was based on the hypothesis that 6 months of EAW + SCES would augment motor recovery compared to only 6 months of EAW. Furthermore, the addition of RT may result in increased muscle size and number of motor units, which could subsequently enhance motor recovery in persons with SCI.

The EAW training sessions lasted 60 min for each participant throughout the duration of the study. An additional 60 min of task-specific training was added to training sessions following implantation for each participant. Testing measurements were taken at baseline [BL], post-intervention 1 [P1] six months after BL, and post-intervention 2 [P2] twelve months after BL.

### 2.3. Temporary Implantation

The participant was maintained in the prone position while temporary implantation was completed in a minor procedure room. Consent was obtained from each participant, and an anesthesia pre-op evaluation was completed before the implantation procedure. During implantation, IV access was established by a nurse, and vital signs (standard ASA monitors including noninvasive blood pressure every 5 min, pulse oximetry, continuous EKG, and end tidal CO2 from a nasal cannula) were continuously monitored throughout the procedure. Loss of resistance technique was utilized by the operating surgeon to access the epidural space via 14-gauge epidural needles with X-ray assistance. Once access was gained, the leads were placed, and configurations were tested, with visible contractions of the paralyzed muscles indicating successful placement. The lead implantation was overseen by a representative from Medtronic. Live fluoroscopy was used to place leads on either side of the midline, confirming posterior epidural placement. Lead position was further optimized after motor stimulation testing. Once the location of the leads was finalized, needles were removed from the epidural space, and tape and glue were used to secure the electrodes to the skin. After implantation, spinal mapping, measured by EMG, was conducted for 3 days beginning on the same day of implantation or the following day [13,20].

### 2.4. Permanent Implantation

Permanent implantation was performed 3–4 weeks after temporary implantation, when activation of the lumbosacral segments was confirmed, and consent was obtained from the participant again. Permanent implantation consisted of two 8-electrode arrays of Vectris leads [13,20]. An anesthesiologist was responsible for IV sedation for permanent implantation, and standard ASA monitoring of vital signs was followed again. The same loss-of-resistance technique with X-ray assistance was utilized by the operating surgeon to access the epidural space via 14-gauge epidural needles again. Once again, configurations were tested, with visible contractions of the paralyzed muscles indicating successful placement in the epidural space [13,20].

The operating surgeon made a vertical incision in the lateral lower back between the 12th rib and the iliac crest of the hip, then created a pocket to place the pulse generator between the skin and muscle. Non-absorbable 0 Monocryl sutures were used to secure the leads to the interspinal ligament and/or lumbodorsal fascia. Then, the leads were connected to the Medtronic Intellis battery in the pocket of tissue created by the surgeon. Impedances were checked after hemostasis was complete and irrigation applied. The wound was sealed in 2–3 layers with 2-0 and 3-0 Vicryl sutures. The outermost opening was treated with Derma-bond, occlusive dressings, and tape for sealing. The participant was given the option of an abdominal binder for comfort if they desired. Antibiotics were prescribed for 5 days. At 7–10 days after permanent implantation, all bandages were removed, as long as recovery was complete. The incision sites were regularly inspected throughout the first month after the implantation. Participants were instructed to abstain from any strenuous physical activity for 3–4 weeks following implantation. The fourth week without physical activity is when spinal mapping was performed [13,20].

### 2.5. Spinal Mapping

Two published reports previously explained the SCES–spinal mapping in detail [21,22]. Briefly, spinal mapping was immediately performed following temporary (3–5 days) and permanent (2–3 weeks) implantation [21,22]. Various parameters (electrode configurations, pulse widths, and stimulation frequencies) were tested to elicit many specific functions. Spinal mapping was performed after the placement of EMG electrodes bilaterally on major muscle groups in a supine position. A frequency of 1 Hz was selected, and the current was gradually ramped at different pulse durations of 250, 500, 750, and 1000 µs. The minimum current (1–14 mA) was applied during mapping to evoke motor activity in the target muscles. Different electrode configurations (anode–cathodes) were tested to determine the most appropriate combinations that may elicit either tonic or rhythmic activation to different muscle groups. From this process, optimal stimulation parameters were determined for each participant and then were re-tested in a standing position using a standing frame at different frequencies (10–60 Hz). The amplitude was then adopted for EAW training with the goal of ensuring smooth stepping and avoiding any jerky movements. We sought participants’ feedback to modify the SCES amplitude during EAW. Spinal mapping was initially conducted with the goal of achieving standing within six months. After P1, mapping was then designed to attain both standing and stepping [20,21,22].

### 2.6. Interventions

#### 2.6.1. Exoskeleton-Assisted Walking (EAW)

Before each training session, the research team assisted the participant into the EAW device while seated, starting at the feet and moving proximally [6,12]. The EAW training schedule was three days per week for 12 months. The EAW software was adjusted based on the needs and progress of each participant. Exoskeleton straps were fastened but checked to make sure they were not too tight to avoid discomfort or inducing episodes of autonomic dysreflexia [6]. Participants started EAW with a roller walker until they gained the ability to shift their weight anterolaterally in a standing position to initiate stepping. The next step was to move to pro-step+ mode, first with a roller walker, then with Canadian crutches roughly 4 weeks later.

In the first 6 months, participants were encouraged to activate SCES for walking using bilateral Canadian crutches. Exoskeleton assistance was set at 100% fixed assistance, and the assistance was gradually lowered by 5% increments until the lowest tolerable level was reached [12]. The decision to reduce the assistance was determined by the ability of the participant to meet an arbitrary threshold of 80% without cueing over a 10 m distance. After dropping the assistance, the EAW was adjusted to the slow mode feature. This feature offered a 2.4 s delay before passively moving the limb. The exoskeleton would beep, indicating that the person failed to move his leg, and automatically advanced the leg. Participants were cued to step by two different buzzers that notified them to complete weight shifting and to initiate stepping. A researcher using two digital counters [one for each leg] would count the number of steps that were actively completed by the participants. The number of active steps was then divided by the total number of steps over a 10 m walking distance. A decision would be made to drop the EAW assistance by 5% if the cut-off was equal to or greater than 80%. The duration of each training session was limited to 60–90 min including fitting, rests, and walking time. Research staff were present throughout each session for guidance and support as well as monitoring of vital signs (heart rate, blood pressure, SPO_2_) every 5 min [6,12]. The fixed assistance was preceded by adaptive assistance for 10–15 min at the beginning of each session to overcome initial stiffness, muscle spasms, and to offer a warm-up for each participant.

EAW was shifted completely to adaptive-assistance mode for the remaining six months of the trial. Adaptability allows the exoskeleton to adjust assistance levels as needed, depending on the participant’s performance. The use of adaptive mode was based on previous findings that suggested enhancement of EAW performance with different neuromodulation techniques [16,20]. The exoskeleton provided support that could range from 0 to 100%, with 100% indicating maximum support for ambulation from the system [12]. Data acquisition sheets for EAW performance per session and for monitoring vital signs throughout the session are included as Supplementary Materials (Supplementary Figure S1).

EAW data were primarily captured using the Ekso Pulse online portal (pulse.eksobionics.com). Ekso Pulse is an online platform interface for monitoring the progress of each participant over the entire session (Supplementary Figure S1). The data were then electronically transmitted and synchronized via cellular technology and stored in a cloud-based system. The data captured from the Ekso Pulse included steps per minute, EAW assistance levels, % minimum assistance walking speed, and distance. Each data point represents an average over a 30 s interval.

#### 2.6.2. Task-Specific Training

Once permanent implantation was complete, a 60 min task-specific training period was added to the participant’s regular training sessions, including sit-to-stand activities and Romanian deadlifts while using a walker or standing frame. The purpose of the standing frame was to promote trunk control by allowing participants to focus on hip extension with their knees locked. After demonstrating proficiency in the standing frame, the task-specific training was moved to parallel bars and finally the standard walker. Previous work describes the need for two research assistants in facilitating trunk control and independent standing during this training [13,20].

### 2.7. Statistical Analysis

#### Exoskeleton Training Performance

Training data were collected by the Ekso and extracted from the Ekso Pulse online portal (https://pulse.eksobionics.com/). Weekly averages (2–3 sessions) were then calculated in Excel sheets for the purpose of analyzing the progression of EAW performance over the course of the 12-month trial. Data were further split into the first 6 months and the remaining 6 months to highlight how varying the assistance mode (fixed vs. adaptive) influenced EAW + SCES performance.

Descriptive data were then grouped by training period into 12-week intervals to further gain insights on the training adaptations per quarter (Q; every 3 months) of the trial. The 12-week periods of training are identified as the first quarter (Q1), second quarter (Q2), third quarter (Q3), and the fourth quarter (Q4).

## 3. Results

**Figure 1:**
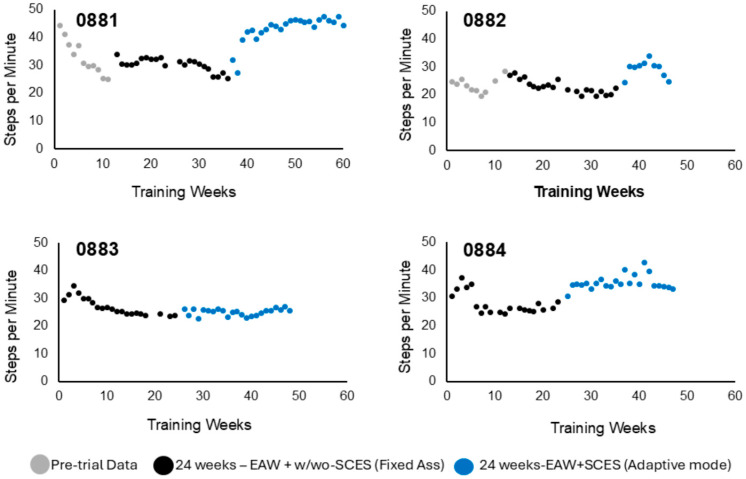
Weekly training progression of EAW steps per minute for the four participants. Gray data points in 0881 and 0882 represent pre-trial data from 12 weeks of EAW prior to SCES implantation, black dots represent the 24 weeks of training after BL testing, and blue dots represent the 24 weeks of training after P1 testing. Three out of the four participants demonstrated a noticeable increase in steps per minute (blue dots) when the EAW assistive mode changed from fixed assistance (black dots) to adaptive mode (blue dots). In participant 0883, the addition of the SCES + EAW–adaptive mode did not influence steps per minute (blue dots) compared to the first 6 months without SCES + EAW–fixed mode. Note that each data point represents the average of 2–3 sessions per week. SDs were omitted for clarity purposes.

**Figure 2:**
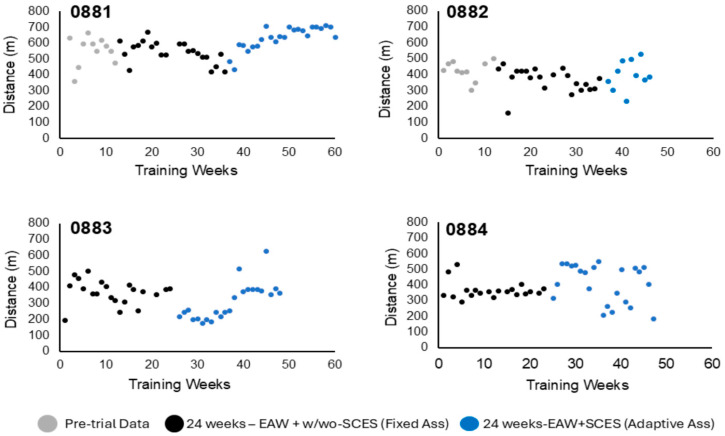
Weekly training progression of EAW distance for the four participants. Gray data points in 0881 and 0882 represent pre-trial data from 12 weeks of EAW prior to SCES implantation, black dots represent the 24 weeks of training after BL testing, and blue dots represent the 24 weeks of training after P1 testing. Two participants (0881 and 0882) noticed a decline in the distance covered towards the end of the first 24 weeks (EAW–fixed assistance + SCES). This may be explained by the fact that EAW assistance was decreased to 45% and 50% for 0881 and 0882, respectively. However, the four participants noted improvement in the distance after switching the assistance to adaptive mode during EAW + SCES. Note that each data point represents the average of 2–3 sessions per week. SDs were omitted for clarity purposes.

**Figure 3:**
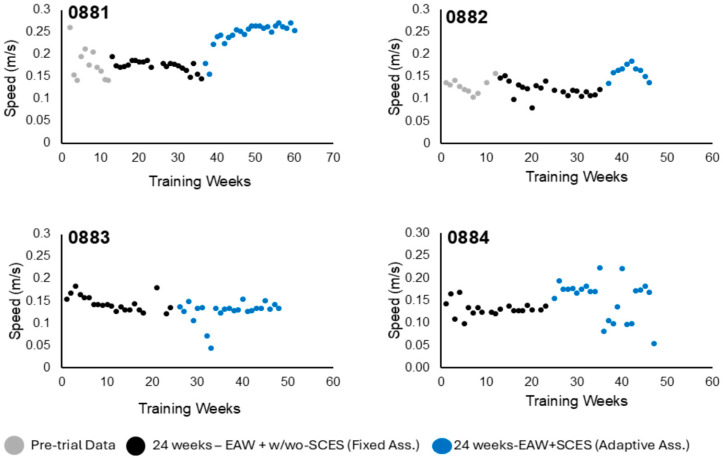
Weekly training progression of EAW speed (m/s) for the four participants. Gray data points in 0881 and 0882 represent pre-trial data from 12 weeks of EAW prior to SCES implantation, black dots represent the 24 weeks of training after BL testing, and blue dots represent the 24 weeks of training after P1 testing. Adaptive assistance managed to increase the EAW + SCES speed in three participants without noticeable changes in 0883 compared to the fixed assistance. SDs were omitted for clarity purposes.

The physical characteristics of all participants are listed in Table 1. Three participants [0881, 0882, and 0884] were randomized into the EAW + SCES + RT group, and the other participant [0883] was randomized into the EAW + SCES + no RT group. Due to this allocation, participant 0883 was implanted 6 months after BL. Two participants (0881 and 0882) were implanted prior to BL, and one participant (0884) was implanted after BL. This discrepancy stemmed from the fact that participants 0881 and 0882 carried over from a previous trial and had an additional 12 weeks of EAW training data without SCES compared to the other two participants. The pre-trial data are included in Figure 1, Figure 2 and Figure 3 for the purpose of visualization and did not appear to influence EAW performance during the 6-months of fixed assistance mode.

During permanent implantation under general anesthesia, participant 0883 experienced greater than expected blood loss. Following completion of the procedure, he was admitted overnight for observation and was then discharged on the following day with no sequelae. Participant 0882 withdrew prior to completion of the trial after developing COVID-19, followed by failure to comply with the study timeline.

### 3.1. EAW Performance

Supplemental Figures S2–S5 present the EAW performance frequency for each participant across the entire course of the study. Table 3 presents the outcomes of SCES spinal mapping, the configurations, and the stimulation parameters across the two phases of the trial. Table 4 demonstrates the average EAW performance across the entire trial. Finally, Table 5 presents the EAW performance based on the mode of assistance (fixed vs. adaptive).

#### 3.1.1. Steps per Minute

**Figure 4:**
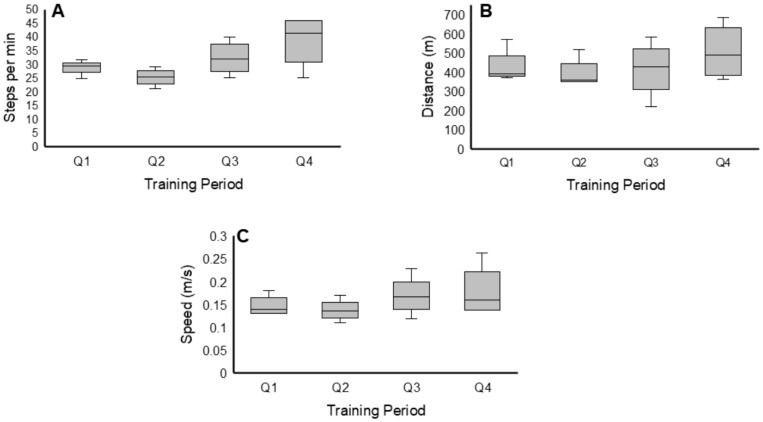
Boxplots displaying the mean ± SD of EAW walking outcomes during training throughout the trial for the entire group. Measurements were captured and are displayed over 12-week intervals for this analysis, as listed along the x-axis (Q1, Q2, Q3, Q4). The first two quarters (Q1 and Q2) represent the average of the data during fixed assistance, and the last two quarters (Q3 and Q4) represent the average of the data during adaptive assistance. (**A**): Steps per minute, (**B**): distance (m), (**C**): speed (m/s). Despite the noticeable increase following Q3 and Q4 in EWA parameters, the data did not attain statistical significance, as highlighted in the results section.

There were clear positive trends in steps per minute observed throughout training (Figure 1). This trend is demonstrated by the mean steps per minute across training periods [Q1: 29.0± 3.0, Q2: 25.0 ± 3.0, Q3: 32.0 ± 6.0, Q4: 38.0 ± 10] (Figure 4). This represents a 31% and 52% increase in the steps per minute during Q4 compared to Q1 and Q2, respectively. Participant 0881 displayed a drastic improvement in steps per minute from BL training to P1 (Figure 1). Participants 0882 and 0884 exhibited a slight increase in steps per minute in the final 6 months of training.

#### 3.1.2. Walking Distance

Mean results indicated a positive training effect on walking distance (m) throughout the trial [Q1: 431.0 ± 93.0, Q2: 396.0 ± 82.0, Q3: 415.0 ± 151, Q4: 506 ± 151] (Figure 4). Participant 0881 exhibited a slight increase in the final 6 months of training (Figure 2). No other participant displayed a positive trend throughout training, as measured by walking distance.

#### 3.1.3. Walking Speed

A slight increase in walking speed (m/s) across the training period was also identified [Q1: 0.15 ± 0.02, Q2: 0.14 ± 0.02, Q3: 0.17 ± 0.05, Q4: 0.18 ± 0.06] (Figure 4). A recognizable increase in walking speed was observed for participant 0881 in the final 6 months of training (Figure 3). Meanwhile, a slight increase in walking speed was noted for participant 0882, and no increase was seen for participant 0884 from BL training to P1 training. Participant 0883 did not display any positive trends throughout training for any of the variables. Distributions for walking outcomes for the entire group across the study are illustrated in Figure 4.

### 3.2. Exoskeleton Assistance

**Figure 5:**
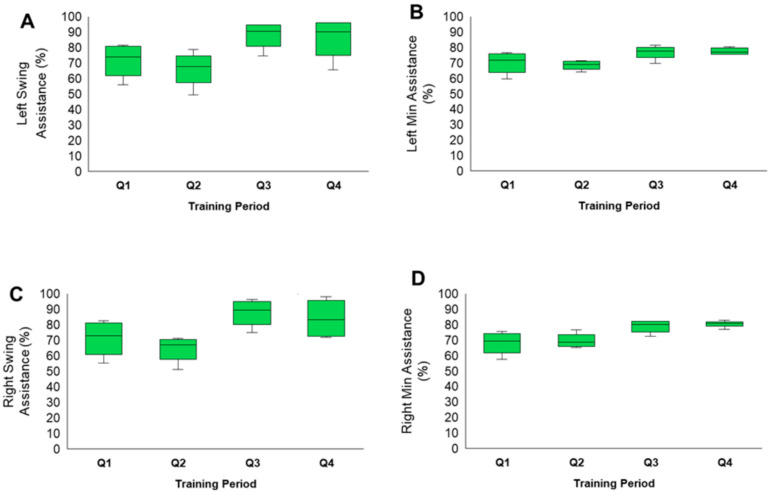
Boxplots displaying the mean ± SD of EAW assistance levels for the entire group throughout the trial. Measurements were captured and are displayed over 12-week intervals, as listed along the x-axis (Q1, Q2, Q3, Q4). (**A**): Left swing assistance, (**B**): left minimum assistance, (**C**): right swing assistance, (**D**): right minimum assistance. All assistance levels are presented as a percentage and were taken directly from the exoskeleton after each training session.

**Figure 6:**
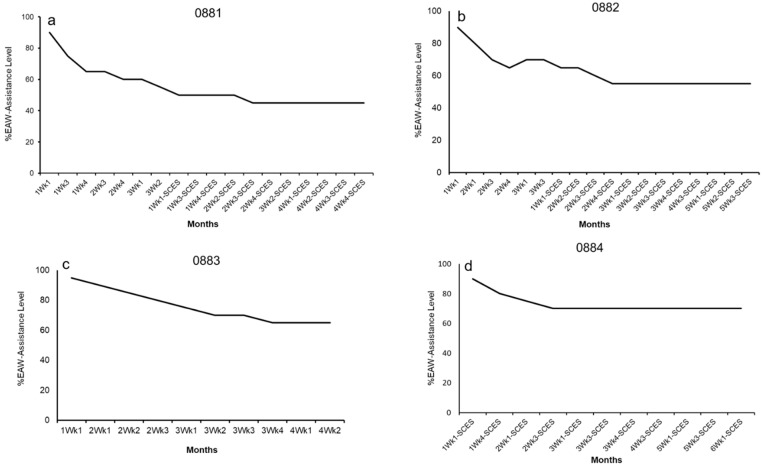
Training progression of %EAW assistance out of maximum assistance (100% fixed assistance) during the fixed assistance EAW mode for participant (**a**) 0881, (**b**) 0882, (**c**) 0883, and (**d**) 0884. This was based on performing a 10 m walk test to measure the number of steps that were actively completed by each participant. A decision to drop the EAW assistance was based on the ability of the participant to actively complete 80% of the total number of steps. The x-axis reflects the timeline at which the EAW–fixed assistance was reduced for each participant. All participants attained a plateau level at which a decrease in EAW assistance was no longer possible.

Distributions for all measured assistance levels (left swing assistance, left minimum assistance, right swing assistance, right minimum assistance) are displayed in Figure 5. All measures indicate a decrease in assistance levels from Q1 to Q2, with an increase from Q2 to Q3. The Q1 and Q2 periods of training consisted of EAW training sessions with warm-up periods of adaptive assistance for only 10–15 min, followed by reduced fixed assistance for the remaining duration of the session. Figure 6 highlights the progression of the EAW–fixed assistance based on the 80% threshold during the initial 6 months of the trial for all participants.

Assistance levels were mostly maintained in adaptive mode only during Q3 and Q4 training periods. Finally, adaptive assistance was measured as an average of 2–3 weeks after BL, either prior to or after P1 and prior to P2. This was carried out to determine the effects of adaptive assistance on EAW parameters throughout the trial (Figure 5). The levels of assistance provided to participants in adaptive mode did not change throughout the study, but the range did noticeably shrink.

## 4. Discussion

The current work examined the longitudinal synergistic hybrid potential of percutaneous SCES with EAW over 12 months in persons with motor complete or complete SCI. Two modes of robotic assistance were implemented over the course of the trial. The first is brief adaptive assistance followed by fixed assistance for the first 6 months. This is followed by adaptive or variable assistance mode only for the remaining 6 months. The overall goal is to determine whether SCES can augment EAW performance, as measured by steps per minute, distance, percentage minimum assistance, and speed after SCI. The findings suggest an association between SCES and augmented EAW performance, especially during adaptive-assistance mode. Minimum assistance is likely to increase during the adaptive mode compared to fixed assistance. However, this is paralleled by increases in steps per minute, walking distance, and speed in persons with SCI.

Despite limited improvements in the participant, 0883 followed a similar pattern of EAW assistance throughout the trial [6 months of fixed assistance followed by 6 months of adaptive assistance]. EAW performance did not change in 0883 following the alteration of the mode of assistance or implantation of SCES compared to the other three participants. This may suggest a closed-loop human–machine interface between SCES and EAW. The use of SCES may have enhanced the peripheral afferent nervous system to sense the changes in EAW assistance and provided sensory flux to increase the rate of motor units firing during the swing phase, especially with the limited supraspinal control in our participants. Although not tested, it was previously established that afferent input related to loading or hip-joint position plays an important role in controlling motor behavior during locomotion [23].

### 4.1. EAW and Hybrid Application

Prior work highlighted the potential benefits of using a robotic exoskeleton to restore overground ambulation, increasing the level of physical activity and subsequently quality of life in persons with SCI and in other clinical populations [1,2,3,4,5,6,24,25]. Recently, a robotic exoskeleton was recognized as an important neuro-prosthesis that may effectively modulate afferent feedback and enhance gait re-education in persons with SCI [8,9,10,11]. A major advantage of using a robotic exoskeleton is the capacity to reduce metabolic cost during overground ambulation compared to other gait re-education interventions [7]. This is likely to reduce muscle fatigue and allow participants with SCI to generate unlimited repetitions of task-specific movements, which is an important concept of neuroplasticity. Therefore, a robotic exoskeleton has been recommended as an alternative platform compared to the recognized body-weight-supported treadmill in the restoration of gait re-education after SCI [9,10,11,12,13,20]. However, the effective translation into active overground steps was limited because of the passive nature of forcing the leg limb movements during the gait cycle after SCI.

Previously, the introduction of hybrid patterns facilitated the synergistic integration of electrical stimulation technology with robotic exoskeletons. The rationale was based on the fact that the combination of these techniques may maximize the benefits compared to a single rehabilitation approach. Furthermore, SCES transforms the dormant lumbosacral segments into more active neural circuitries to receptively integrate the sensory flux during EAW performance into segmental motor commands [8,9,10]. A caveat to the hybrid approach is synchronizing the time between both modalities to allow fluency of the movement during the gait cycle in persons with SCI [12,20]. A recent study successfully employed closed-loop spatiotemporal SCES with different robotic platforms like Lokomat, Myosuit, and Clickers [8]. The study highlighted the significance of synergistically applying both technologies (SCES with different robotic techniques) to reduce fatigue and enhance overground ambulation, especially early in rehabilitation and in those with severe SCI [8]. Proof-of-concept studies demonstrated the successful application of a hybrid approach within either open or closed-loop SCES to enhance overground ambulation after SCI [8,18,19,20,,26].

### 4.2. EAW + SCES Application

In the current study, SCES was delivered at either submotor or motor thresholds to ensure synchronization between both rehabilitation technologies [18]. The submotor thresholds (i.e., amplitudes in mA) were primarily determined in supine lying positions before being examined or tweaked during standing or stepping in persons with SCI. It is possible to assume that altering the position from lying to stepping with an exoskeleton may have influenced the level of stimulation during EAW performance. The stimulation thresholds were marginally higher during the adaptive mode compared to the fixed EAW mode in three of the four participants (see Table 1). Optimizing the stimulation parameters (amplitude, pulse duration, and frequency) is a daunting task and highly challenging during task-specific training, similar to standing and stepping. We have attempted to develop a step-by-step procedure on how to configure and optimize the SCES parameters to neuromodulate the lumbosacral segments [21,22]. We also ensured that there was no direct stimulation of the corresponding lumbosacral segments, but rather enabled the lumbosacral segments, as measured by the EMG motor evoked potentials [20,21,22]. Additionally, we intentionally developed strategies that allow either lowering the EAW assistance from maximum assistance to fixed assistance or providing adaptive assistance during stepping [12,17].

### 4.3. Fixed vs. Adaptive-Assistance Mode in Association with SCES

Switching from fixed to adaptive-assistance mode augmented the capacity of SCES on EAW performance (see Table 4). The augmented capacity of SCES was previously documented in four persons with SCI during 50% body -weight-supported treadmill training stepping at 0.36 m/s. SCES increased the rhythmic activities of four different muscle groups compared to therapist-assisted stepping on treadmill training without SCES [18]. The authors noted that proprioceptive enhancement via therapist-assisted treadmill training is noted with the addition of the SCES. Our group previously noted similar findings once the SCES configurations enhanced rhythmic muscle activities during 300 steps of EAW. Compared to non-rhythmic SCES configurations, two participants demonstrated increases in EAW steps per minute and step length [20].

In the current paper, the augmented capacity of SCES on EAW performance during adaptive-assistance mode could be explained by applications of rhythmic SCES configurations, enhancement of the spinal cord central pattern generator, and/or decreased dyssynergia between the agonist and antagonist muscle groups. The study did not measure any of the above assumptions during EAW training sessions. The EAW training lasted for 60 min per session, and it was unlikely to measure these changes over 12 months. However, these measurements were captured during the 10 m walking test or via a dynamometer to measure spasticity of the right knee extensor muscle group only during the BL, P1, and P2 assessment periods [13].

Previous multicenter or randomized clinical trials were only limited to 8–16 weeks of EAW [4,5]. There are limited research findings considering the long-term applications of robotic EAW in persons with SCI [27]. Despite the preliminary nature of our findings, the current 12-month trial of the hybrid application will provide the basis for future clinical trials to examine home-based potential after SCI. This would require the presence of a dedicated and highly trained caregiver to ensure completion of the trial. A recent multi-center cooperative trial demonstrated that the absence of a caregiver may result in limited EAW use over a 16-week period and did not influence the mental component or quality of life compared to the control group [4].

### 4.4. EAW Metrics [Steps per Minute, Speed, and Distance]

Another important point is the number of steps per minute and EAW speed during the entire 60 min session. Twelve weeks of EAW training (gray data) demonstrated a decline in the number of steps per minute, as highlighted in Figure 1. However, the implantation of SCES resulted in maintenance of 25–29 steps per minute and 32–38 steps per minute during fixed and adaptive-assistance modes, respectively. As previously shown, this hybrid approach may augment motor outcomes even in persons with clinically complete SCI [8,11,12,17]. The increase in the number of steps should have been incorporated with measuring EMG activities to highlight potential improvements in neurological motor control.

Edwards et al. reported improvements in walking speed of 0.18–0.43 m/s following 12 weeks of EAW training in persons with incomplete SCI [11]. The study highlighted two important speed thresholds of 0.15 m/s and 0.44 m/s for minimal clinical detectability and for community ambulation, respectively [11]. Our findings indicated that decreasing EAW assistance to a fixed level maintained a speed of 0.14 m/s. However, participants were capable of maintaining 0.16 m/s during adaptive mode with SCES on. An important consideration is that the reported speeds represent the average during a 60 min training session and not during a 10 m walking test.

During the first 6 months, a 10 m walking test was performed at the end of every week to determine whether EAW assistance should or should not be lowered by 10%, similar to the earlier report [12]. This was based on whether participants could achieve 80% active steps without assistance from the robotic exoskeleton with SCES on. Participants in the current study managed to reduce EAW–fixed assistance to 55–70%. This fixed assistance strategy was previously adopted and demonstrated that in participants with SCI, the EAW assistance was successfully decreased to 35% or 55% when using SCES or TSS, respectively [12,17]. Others indicated that robotic variable assistance is more effective in allowing participants to mimic real-life overground stepping without limiting performance, as highlighted for fixed assistance [16]. The statistical discrepancy in EAW–adaptive assistance (see Figure 5) may reflect the extent of variance in the actuator performance between the right and left legs to ensure overground stepping. Therefore, examining EAW performance over the course of 12 months was a highly essential strategy, especially with the rise in the number of future studies likely to examine such a hybrid approach after SCI.

## 5. Limitations

The exploratory nature of the trial and the small sample size (n = 4) limited the power of the analysis and the significance of the results. The limited recruitment may be explained by the 12-month duration of the study, which required long-term commitment. However, our sample size is not different from other well-established clinical trials in the field [28,29,30]. Future clinical trials should consider a 6-month intervention to reduce the burden on the participants, as well as other SCI indications similar to spasticity, autonomic-cardiovascular, and bladder functions. We only studied individuals with complete and motor complete SCI. Magnetic resonance imaging showed that the axonal tissue damage ranged from 80 to 92% [21]. The findings suggested that the four persons experienced discomplete SCI despite their AIS classification with one level of spared sensory zones of partial presentation (see Table 1). The use of MRI is for morphological assessment and should be incorporated with measuring motor-evoked potential tests for functional examination. Furthermore, the participants experienced mild to moderate degrees of spasticity at admission, which is consistent with neurophysiological proof of discompleteness [31]. However, it is unlikely that this may have influenced the augmented capacity of SCES in EAW performance in the current trial.

Participants 0881 and 0882 originally participated in 12 weeks of EAW training prior to SCES implantation. However, the effect of 12 weeks of EAW was modest and did not appear to influence their performance following SCES implantation, as noted in Figure 1, Figure 2 and Figure 3. Additionally, one of the participants, # 0882, did not complete the study, further limiting the available data. Another limitation was the battery life of the robotic exoskeleton. In participants 0883 and 0884, batteries had to be frequently replaced during training sessions, which would create otherwise unscheduled interruptions to EAW sessions.

There was also a failure to measure EMG activities or to account for muscle dyssynergia in actual training sessions during the 12-month training period. Previous trials used assisted treadmill training and measured EMG activities during walking within a confined space. EAW training provided free walking distance, but it is unlikely that the wireless EMG would accurately synchronize the data with the main station after exceeding 10 m. Therefore, we limited our EMG measurements to the 10 m walking distance to ensure accurate synchronization with the main station (data not presented).

## 6. Conclusions

Twelve months of hybrid SCES with EAW resulted in increased steps per minute, distance, and speed during adaptive mode in persons with motor complete or complete SCI. Furthermore, the preliminary report highlighted the synergistic pattern induced by SCES during reduced EAW assistance, especially after altering the assistance mode. Considering the small sample size, the study placed stronger emphasis on the descriptive case report findings of the work to avoid implying hypothesis-driven inferential conclusions. Future studies may warrant a large sample size to test the hypothesis that the adaptive-assistance mode is favorable compared to the fixed mode during EAW. Furthermore, adaptive-assistance mode may provide variable and adjustable levels of assistance based on the motor performance of each participant, which mimics real-life scenarios during walking. These findings may serve as the basis for future studies to consider hybrid integration of exoskeletons with neuromodulation techniques to further enhance the rehabilitation potential after SCI.

## Figures and Tables

**Table 1:** Physical and SCI characteristics of each participant at the time of enrollment in the trial, as well as the lowest level of Ekso-EAW assistance (Ass.) attained during the initial 6 months of training.

ID	Sex	Age (year)	Weight (kg)	Height (cm)	BMI (kg/m^2^)	TSI (year)	NLI	AIS	Classification	Level of Ekso. Ass.	Assistive Device	Weeks Attained **	Amp (mA)-First 6 Month	Amp (mA)-Last 6 Month	MAS at the Time of Admission
0881	M	25	48.6	174.3	16.0	6.0	C8	A	Tetraplegia	45%	Canadian crutches	8	1.1–6	4.6	1+−3
0082	M	36	99.4	182.2	29.9	9.0	T11	B	Paraplegia	50%	Canadian crutches	13	1.6–2.7	2–4	2
0883	M	38	99.0	180.5	30.4	12.0	T6	A	Paraplegia	70%	Standard roller walker	9	No SCES	7	1–2
0884 *	M	54	93.4	182.8	28.0	24.0	T4	A	Paraplegia	70%	Standard roller walker/Canadian crutches	7	6–8	7.9	0–2

Note: *, 0884 used both assistive modes alternatively based on his performance. M: male; BMI: body mass index; TSI: time since injury; NLI: neurological level of injury; AIS: American Spinal Cord Injury Association (ASIA) Impairment Scale. All participants had an AIS exam at the time of admission, with one level of sensory zone of partial preservation; MAS: Modified Ashworth Scale; **, represents the number of weeks required to switch from standard roller walker to Canadian crutches.

**Table 2:** Timeline of the study design, including assessment, mapping, and training for persons with chronic spinal cord injury.

	Assessment Period and Mapping					Training	Assessment Period and Mapping					Training		
Study Events	Baseline Ass.	Temporary Implantation and Mapping	Permanent Implant	Resting Period	Mapping	Training Phase 1	P1 Ass.	Interim Mapping	Permanent Implant	Resting Period	Mapping	Training Phase 2	Training Phase 2	P2 Ass
Group 1 [EAW + SCES + RT]	1 week	3–5 days	Three weeks Later	Three weeks to avoid migration	2–3 weeks	1 h EAW + SCES for 24 weeks + 1 h of task-specific training (sit-to-stand activity) for 24 weeks	1 week	3 weeks	Completed in Phase 1	Completed in Phase 1	Completed in Phase 1	1 h EAW + SCES for 12 weeks + 1 h of task-specific training (sit-to-stand activity) for 12 weeks + 12 weeks of NMES-RT	1 h EAW + SCES for 12 weeks + 1 h of task-specific training (sit-to-stand activity) for 12 weeks + 12 weeks of closed-chain RT using SCES	1 week
Group 2 [EAW+ delayed SCES + noRT]	1 week	3–5 days	Delayed implantation to the second phase of the trial	Not applicable	Not applicable	1 h EAW only for 24 weeks	1 week	Not applicable	After P1	Three weeks to avoid migration	2–3 weeks	1 h EAW + SCES for 12 weeks + 1 h of task-specific training (sit-to-stand activity) for 12 weeks + 12 weeks of PMT	1 h EAW + SCES for 12 weeks + 1 h of task-specific training (sit-to-stand activity) for 12 weeks	1 week
						3 days per week						3–5 days per week		
Weeks	Week 1	Previously completed prior to BL assessment				Week 2–25	Week 26	Week 27–29				Week 30–41	Week 42–53	Week 54
Weeks	Week 1	Week 2	Week 3	Week 4–6	Week 7–9	Week 10–33	Week 34	Week 35–37				Week 38–49	Week 50–61	Week 62
Weeks	Week 1	Week 2				Week 3–26	Week 27		Week 28	Week 31	Week 32–35	Week 36–47	Week 48–59	Week 60
Two phases	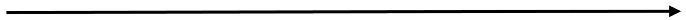							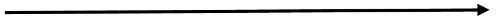						

Ass: assistance; MAS: modified Ashworth scores; weeks attained to switch from walker to Canadian crutches; Amp: submotor or motor threshold amplitude (mA) ranges that were used for 12 months of EAW training. The amplitudes were primarily determined based on supine EMG testing before they were modified for EAW. MAS were reported as the range of scores for the flexor and the extensors of the hip flexors, knee flexors and extensors, and calf muscles. The arrows are depicting the flow of the study during each study phase.

**Table 3:** Configurations and stimulation parameters during the two phases of EAW training for each participant with SCI.

	0881	0882	0883	0884
EAW Training Phase 1 (24 weeks)	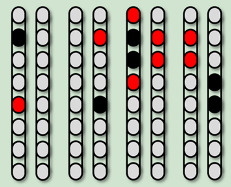	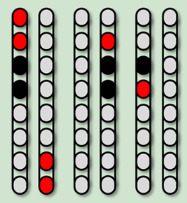	No SCES	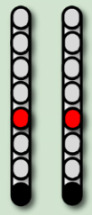
Configurations				
Stimulation Parameters	1.7–3.5 mA, 350–450 µs, 25 Hz	1.6–2.3 mA, 350–450 µs, 20 Hz		6–8 mA, 210 µs, 40 Hz
EAW Training Phase 2 (24 weeks)				
Configurations	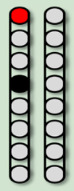	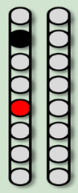	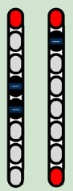	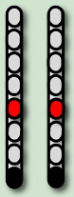
Stimulation Parameters	3.0 mA, 250 µs, 25 Hz	3.5 mA, 250 µs, 30 Hz	7 mA, 300 µs, 60 Hz,	6–8 mA, 210 µs, 40 Hz

Red electrode represents the anode, black anode represents the cathode, gray are inactive electrodes.

**Table 4:** Number of weeks completed for each participant, as well as EAW performance throughout the entire study, as measured by Ekso Pulse software.

Participant ID	Number of Weeks (48 Weeks)	Missed Weeks	Up Time (min)	Walk Time (min)	% Walk Time to up Time	Number of Steps	Distance (m)	Speed (m/s)
0881	46	2	66 ± 7	47 ± 5.7	72 ± 4.5	1727 ± 243	592 ± 83	0.21 ± 0.04
0882	31	17 *	65 ± 14.5	48 ± 19.0	74 ± 9.0	1151 ± 238	380 ± 78.5	0.13 ± 0.02
0883	44	4	56 ± 13.5	42 ± 11.0	74 ± 7.0	1074 ± 319	341 ± 101	0.14 ± 0.02
0084	43	5	56 ± 6.0	45 ± 5.0	81 ± 4.0	1308 ± 328	392 ± 98	0.15 ± 0.04
Average	41 ± 7.0	3.7 ± 2.0	61 ± 6.0	46 ± 3.0	75 ± 4.0	1315 ± 291	426 ± 113	0.16 ± 0.04

* 0082 completed only 31 weeks of the entire trial before he decided to withdraw due to failure to comply with the study timeline.

**Table 5:** Comparison between fixed vs. adaptive (Adap.) modes of EAW-Ass., highlighting the number of weeks completed for each participant as well as EAW performance throughout each level of Ass., as measured by Ekso Pulse software. The highlighted gray areas represent the adaptive mode at different EAW variables.

Participant ID	Number of Weeks		Up Time (min)		Walk Time (min)		% Walk Time to up Time		Number of Steps		Distance (m)		Speed (m/s)	
EAW Ass. Mode	Fixed	Adap.	Fixed	Adap.	Fixed	Adap.	Fixed	Adap.	Fixed	Adap.	Fixed	Adap.	Fixed	Adap.
0881	21	25	70 ± 8	62 ± 2 *	52 ± 5	43 ± 2 *	74 ± 5	70 ± 3 *	1604 ± 183	1830 ± 242 *	550 ± 63	627 ± 83 *	0.18 ± 0.01	0.24 ± 0.04 *
0882	21	10	70 ± 14	56 ± 11 *	52 ± 12	42 ± 9 *	74 ± 10	74 ± 10	1122 ± 218	1213 ± 277	370 ± 72	400 ± 91	0.12 ± 0.02	0.16 ± 0.02 *
0883	20	24	58 ± 9	55 ± 16	42 ± 8	42 ± 13	72 ± 9	76 ± 6	1167 ± 244	996 ± 357 ^x^	371 ± 77	316 ± 113 ^x^	0.15 ± 0.02	0.13 ± 0.02 *
0084	20	23	59 ± 5	54 ± 6 *	46 ± 3	45 ± 6	79 ± 4	82 ± 4 *	1229 ± 182	1376 ± 408 *	369 ± 55	413 ± 123 ^x^	0.13 ± 0.02	0.16 ± 0.04 *
**Average**	**20.5 ± 1**	**20.5 ± 7**	**64 ± 7**	**57 ± 4**	**48 ± 5**	**43 ± 1**	**75 ± 3**	**76 ± 5**	**1281 ± 220**	**1354 ± 353**	**415 ± 90**	**439 ± 133**	**0.15 ± 0.03**	**0.17 ± 0.04**

* Independent *t*-tests revealed statistically significant differences between adaptive and fixed modes of assistance (*p* < 0.05). x, a trend towards statistical significance; *p* = 0.06–0.07.

## Data Availability

Data will be shared by the lead contact author [Ashraf Gorgey (ashraf.gorgey@va.gov)] upon request and after obtaining necessary approval from the local research office.

## Supplementary Materials

The following supporting information can be downloaded at: https://www.mdpi.com/article/10.3390/life16010077/s1, Supplementary Figure S1. Representative figure of Ekso Pulse, which is a cloud-based analytics platform located at https://pulse.eksobionics.com/. The platform is designed to monitor the progress of the Ekso exoskeleton and allow clinicians-researchers to view and analyze the reports and eventually adjust the plans. The platform provides the subject ID, date of the session, walk time, minimum assistance or swing assistance, session stats, etc. The exoskeleton automatically records and stores the data during the actual session and then synchronizes via Wi-Fi wireless technology to the Ekso’s secure cloud storage. The data from each single session can be simply exported to excel sheet for analysis. Supplemental Figures S2–S5. The distribution of the frequency of occurrence (weeks; y-axis) of the up time (min), walk time (min), % walk time/up time), number of steps, distance (m), and speed (m/s), plotted on the x-axis, across the entire 48 weeks for participants 0881 (Supplementary Figure S1), 0882 (Supplementary Figure S2), 0883 (Supplementary Figure S3), 0884 (Supplementary Figure S4). Each frequency point represents an average of 2–3 sessions across the entire week. The solid black line represents a bell-shaped curve that helps determine the skewness of the data across the entire course of the study. Supplementary Table S1. EAW data collection sheets that were used every session for the entire study.

## References

[1] Gorgey A.S., Sumrell R., Goetz L.L. (2019). Exoskeletal assisted rehabilitation after spinal cord injury. Atlas of Orthoses and Assistive Devices.

[2] Gil-Agudo Á., Megía-García Á., Pons J.L., Sinovas-Alonso I., Comino-Suárez N., Lozano-Berrio V., Del-Ama A.J. (2023). Exoskeleton-based training improves walking independence in incomplete spinal cord injury patients: Results from a randomized controlled trial. J. Neuroeng. Rehabil..

[3] Tan K., Koyama S., Sakurai H., Teranishi T., Kanada Y., Tanabe S. (2021). Wearable robotic exoskeleton for gait reconstruction in patients with spinal cord injury: A literature review. J. Orthop. Translat..

[4] Spungen A.M., Dematt E.J., Biswas K., Jones K.M., Mi Z., Snodgrass A.J., Morin K., Asselin P.K., Cirnigliaro C.M., Kirshblum S. (2024). Exoskeletal-Assisted Walking in Veterans with Paralysis: A Randomized Clinical Trial. JAMA Netw. Open.

[5] Baunsgaard C.B., Nissen U.V., Brust A.K., Frotzler A., Ribeill C., Kalke Y.-B., León N., Gómez B., Samuelsson K., Antepohl W. (2018). Gait training after spinal cord injury: Safety, feasibility and gait function following 8 weeks of training with the exoskeletons from Ekso Bionics. Spinal Cord.

[6] Lester R.M., Gorgey A.S. (2018). Feasibility of robotic exoskeleton ambulation in a C4 person with incomplete spinal cord injury: A case report. Spinal Cord Ser. Cases.

[7] Shepertycky M., Burton S., Dickson A., Liu Y.F., Li Q. (2021). Removing energy with an exoskeleton reduces the metabolic cost of walking. Science.

[8] Hankov N., Caban M., Demesmaeker R., Roulet M., Komi S., Xiloyannis M., Gehrig A., Varescon C., Spiess M.R., Maggioni S. (2025). Augmenting rehabilitation robotics with spinal cord neuromodulation: A proof of concept. Sci. Robot..

[9] Ivanenko Y., Shapkova E.Y., Petrova D.A., Kleeva D.F., Lebedev M.A. (2023). Exoskeleton gait training with spinal cord neuromodulation. Front. Hum. Neurosci..

[10] He Y., Xu Y., Hai M., Feng Y., Liu P., Chen Z., Duan W. (2024). Exoskeleton-Assisted Rehabilitation and Neuroplasticity in Spinal Cord Injury. World Neurosurg..

[11] Edwards D.J., Forrest G., Cortes M., Weightman M.M., Sadowsky C., Chang S.-H., Furman K., Bialek A., Prokup S., Carlow J. (2022). Walking improvement in chronic incomplete spinal cord injury with exoskeleton robotic training (WISE): A randomized controlled trial. Spinal Cord.

[12] Gorgey A.S., Gill S., Holman M.E., Davis J.C., Atri R., Bai O., Goetz L., Lester D.L., Trainer R., Lavis T.D. (2020). The feasibility of using exoskeletal-assisted walking with epidural stimulation: A case report study. Ann. Clin. Transl. Neurol..

[13] Gorgey A.S., Trainer R., Khalil R.E., Deitrich J., Rehman M.U., Goetz L.L., Lester D., Klausner A., Peterson C.L., Lavis T. (2025). Epidural Stimulation and Resistance Training (REST-SCI) for Overground Locomotion After Spinal Cord Injury: Randomized Clinical Trial Protocol. J. Clin. Med..

[14] Dunkelberger N., Berning J., Schearer E.M., O’Malley M.K. (2023). Hybrid FES-exoskeleton control: Using MPC to distribute actuation for elbow and wrist movements. Front. Neurorobotics.

[15] Stauffer Y., Allemand Y., Bouri M., Fournier J., Clavel R., Metrailler P., Brodard R., Reynard F. (2009). The WalkTrainer--a new generation of walking reeducation device combining orthoses and muscle stimulation. IEEE Trans. Neural Syst. Rehabil. Eng..

[16] Gad P., Gerasimenko Y., Zdunowski S., Turner A., Sayenko D., Lu D.C., Edgerton V.R. (2017). Weight Bearing Over-ground Stepping in an Exoskeleton with Non-invasive Spinal Cord Neuromodulation after Motor Complete Paraplegia. Front. Neurosci..

[17] Sutor T.W., Ghatas M.P., Goetz L.L., Lavis T.D., Gorgey A.S. (2022). Exoskeleton Training and Trans-Spinal Stimulation for Physical Activity Enhancement After Spinal Cord Injury (EXTra-SCI): An Exploratory Study. Front. Rehabil. Sci..

[18] Minassian K., Hofstoetter U.S. (2016). Spinal Cord Stimulation and Augmentative Control Strategies for Leg Movement after Spinal Paralysis in Humans. CNS Neurosci. Ther..

[19] Krenn M.J., White J.M., Stokic D.S., Tansey K.E. (2023). Neuromodulation with transcutaneous spinal stimulation reveals different groups of motor profiles during robot-guided stepping in humans with incomplete spinal cord injury. Exp. Brain Res..

[20] Gorgey A.S., Trainer R., Sutor T.W., Goldsmith J.A., Alazzam A., Goetz L.L., Lester D., Lavis T.D. (2023). A case study of percutaneous epidural stimulation to enable motor control in two men after spinal cord injury. Nat. Commun..

[21] Alazzam A.M., Ballance W.B., Smith A.C., Rejc E., Weber K.A., Trainer R., Gorgey A.S. (2024). Peak Slope Ratio of the Recruitment Curves Compared to Muscle Evoked Potentials to Optimize Standing Configurations with Percutaneous Epidural Stimulation after Spinal Cord Injury. J. Clin. Med..

[22] Venigalla S., Rehman M.U., Deitrich J.N., Trainer R., Gorgey A.S. (2024). MRI Spinal Cord Reconstruction Provides Insights into Mapping and Migration Following Percutaneous Epidural Stimulation Implantation in Spinal Cord Injury. J. Clin. Med..

[23] Dietz V. (2002). Proprioception and locomotor disorders. Nat. Rev. Neurosci..

[24] Copilusi C., Dumitru S., Dumitru N., Geonea I., Mic C. (2024). An Exoskeleton Design and Numerical Characterization for Children with Duchenne Muscular Dystrophy. Bioengineering.

[25] Lee Y.-H., Ko L.-W., Hsu C.-Y., Cheng Y.-Y. (2023). Therapeutic Effects of Robotic-Exoskeleton-Assisted Gait Rehabilitation and Predictive Factors of Significant Improvements in Stroke Patients: A Randomized Controlled Trial. Bioengineering.

[26] Liu P., Cheng Y., Xu Z., Li X., Chen Z., Duan W. (2025). Spatiotemporal spinal cord stimulation with real-time triggering exoskeleton restores walking capability: A case report. Ann. Clin. Transl. Neurol..

[27] Zieriacks A., Aach M., Brinkemper A., Koller D., Schildhauer T.A., Grasmücke D. (2021). Rehabilitation of Acute Vs. Chronic Patients with Spinal Cord Injury with a Neurologically Controlled Hybrid Assistive Limb Exoskeleton: Is There a Difference in Outcome?. Front. Neurorobotics.

[28] Gill M.L., Grahn P.J., Calvert J.S., Linde M.B., Lavrov I.A., Strommen J.A., Beck L.A., Sayenko D.G., Van Straaten M.G., Drubach D.I. (2018). Neuromodulation of lumbosacral spinal networks enables independent stepping after complete paraplegia. Nat. Med..

[29] Darrow D., Balser D., Netoff T.I., Krassioukov A., Phillips A., Parr A., Samadani U. (2019). Epidural Spinal Cord Stimulation Facilitates Immediate Restoration of Dormant Motor and Autonomic Supraspinal Pathways after Chronic Neurologically Complete Spinal Cord Injury. J. Neurotrauma.

[30] Angeli C.A., Boakye M., Morton R.A., Vogt J., Benton K., Chen Y., Ferreira C.K., Harkema S.J. (2018). Recovery of Over-Ground Walking after Chronic Motor Complete Spinal Cord Injury. N. Engl. J. Med..

[31] Wahlgren C., Levi R., Amezcua S., Thorell O., Thordstein M. (2021). Prevalence of discomplete sensorimotor spinal cord injury as evidenced by neurophysiological methods: A cross-sectional study. J. Rehabil. Med..

