# Supplementary material for: Hybrid 12-Month Exoskeleton Training with Percutaneous Epidural Stimulation After Spinal Cord Injury
Source: Life (Basel). 2026 Jan 4;16(1):77. doi: 10.3390/life16010077 (PMC12843262; doi:10.3390/life16010077)
Supplement: Supplementary file 1 [file life-16-00077-s001.zip › Supplementary Table S1. EKSO_TRAINING_SHEETs_SuppLDoc.pdf]

FORM 5: CLINICAL DATA COLLECTION

Date: ____________________

Patient Name: ______________________________ Patient ID: ______________ Age: ________

Type of Injury: ______________________________ Date Of Injury (DOI): ______________

Session Dates:					
**EKSO SETUP**					
(W) Hip Width / (A) Hip Abduction Value	W / A	W / A	W / A	W / A	W / A
(U) Upper Leg / (L) Lower Leg	U / L	U / L	U / L	U / L	U / L
Right Ankle Settings [(S)Spring 1-4)/(D)Degree -3,-2,-1,0,1,2]	S / D	S / D	S / D	S / D	S / D
Left Ankle Settings [(S)Spring 1-4)/(D)Degree -3,-2,-1,0,1,2]	S / D	S / D	S / D	S / D	S / D
Hip Free: (R) Rotation / (A) Abduction (Y/N)	R / A	R / A	R / A	R / A	R / A
Torso / Hip / Tibial Shims / Pads					
Foot Binding Pad / Shoe Lift (L) Left/(R) Right	L / R	L / R	L / R	L / R	L / R
Crutch / Rolling Walker / Cane Height Settings					
Arm Sling Used (Y/N)					
**SOFTWARE SETTINGS**					
(L) Step Length / (H) Step Height	L / H	L / H	L / H	L / H	L / H
(SW) Swing Time / (ST) Stand Time	SW / ST	SW / ST	SW / ST	SW / ST	SW / ST
(K) Knee Flex / (H) Hip Flex	K / H	K / H	K / H	K / H	K / H
Relative Walk Angle					
(F) Forward Target / (L) Lateral Target	F / L	F / L	F / L	F / L	F / L
**ASSISTANCE SETTINGS** *Default Settings					
Type (Bilat*/R. AFF/L. AFF)					
Left Swing Forward Assist (Max*/Adapt/Fixed)					
Right Swing Forward Assist (Max*/Adapt/Fixed)					
Baseline (Default = 5.0)					
Free Leg Stance Support (High*/Med/Low/Off)					
Swing Complete (Fast*/Medium/Slow)					
Heel Strike (HS) Beep (Off*/On)					
**WALK SOFTWARE OPTIONS**					
Crutch / Walker Stand					
Walk Method (First/Active/Pro/Pro+)					
Train (Forward/Lateral/Both/None)					
**DATA RECORDED**					
Walk Time					
Up Time					
Total Steps					
**ASSISTANCE FEEDBACK**	(5 OR 60 STEP)	(5 OR 60 STEP)	(5 OR 60 STEP)	(5 OR 60 STEP)	(5 OR 60 STEP)
Forward Assist (L) Left/(R) Right	L / R	L / R	L / R	L / R	L / R
Min Assist (L) Left/(R) Right	L / R	L / R	L / R	L / R	L / R
Path Assist (L) Left/(R) Right	L / R	L / R	L / R	L / R	L / R
Vitals					

Comments:

DATE:_________________________ **Temp:**_________________________

**Total time:**	BP	HR	RPE
PRE-SEATED			
PRE-STANDING			
POST-STANDING			
POST-STEATED			
TOTAL SESSION TIME:			
**EKSO CLOCK:**			

DATE:_________________________ **Temp:**_________________________

**Total time:**	BP	HR	RPE
PRE-SEATED			
PRE-STANDING			
POST-STANDING			
POST-STEATED			
TOTAL SESSION TIME:			
**EKSO CLOCK:**			

DATE:_________________________ **Temp:**_________________________

**Total time:**	BP	HR	RPE
PRE-SEATED			
PRE-STANDING			
POST-STANDING			
POST-STEATED			
TOTAL SESSION TIME:			
**EKSO CLOCK:**			
