# Supplementary figures and images for: Hybrid 12-Month Exoskeleton Training with Percutaneous Epidural Stimulation After Spinal Cord Injury
Source: Life (Basel). 2026 Jan 4;16(1):77. doi: 10.3390/life16010077 (PMC12843262; doi:10.3390/life16010077)

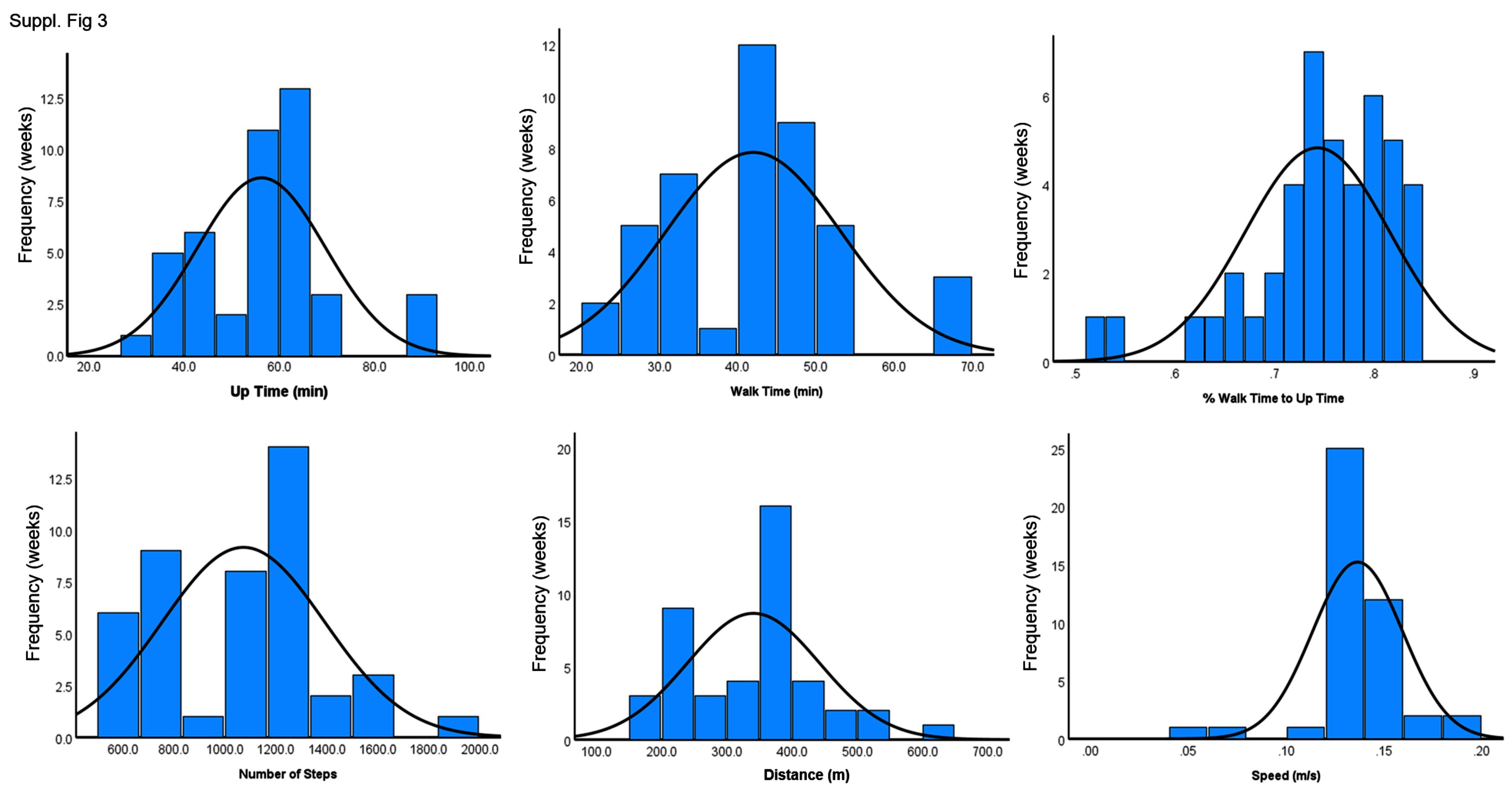

Supplement: Supplementary file 1 [file life-16-00077-s001.zip › 0883M_Supplementary Figure S3.jpg]

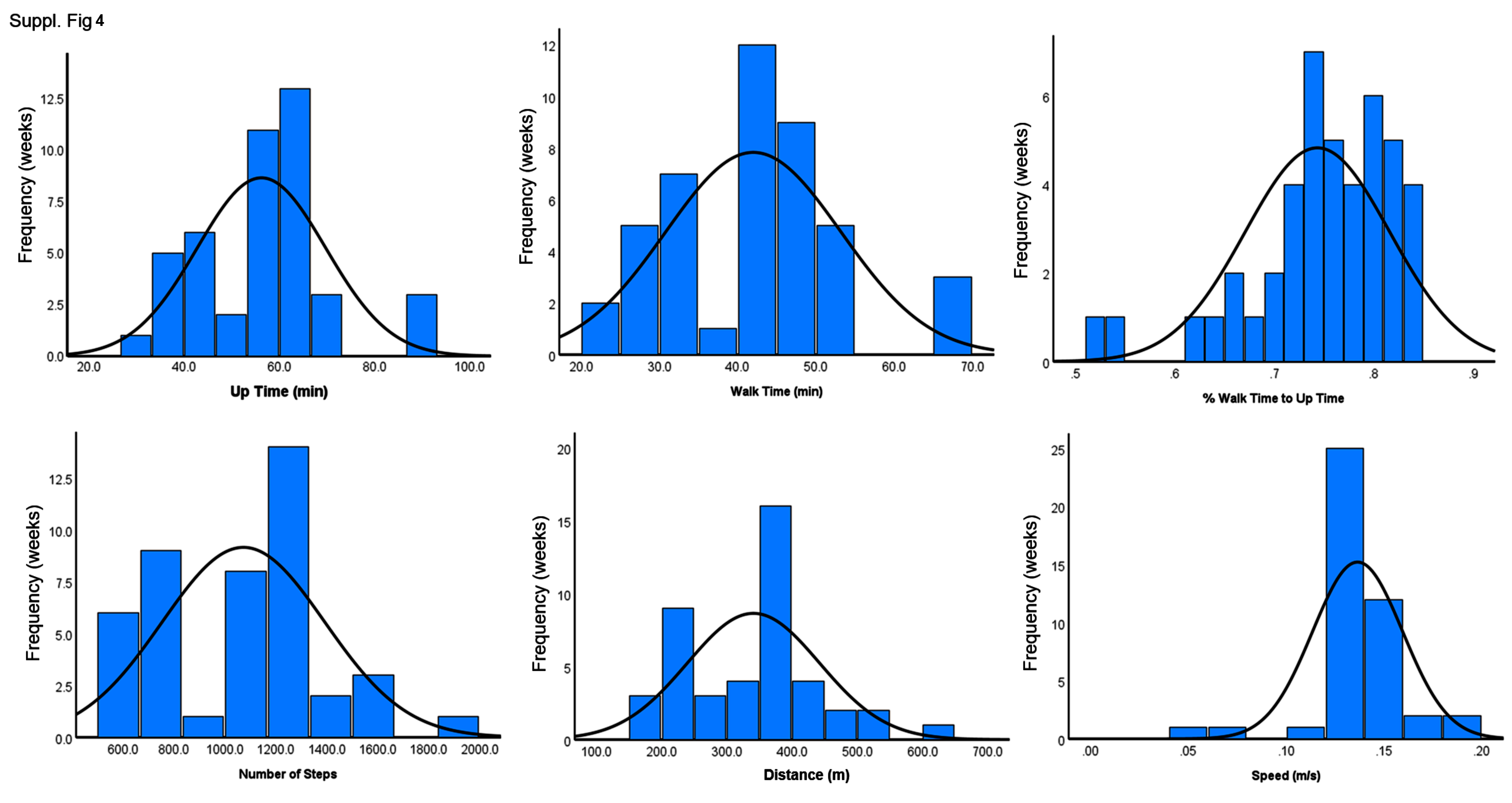

Supplement: Supplementary file 1 [file life-16-00077-s001.zip › 0883M_Supplementary Figure S4.tif]

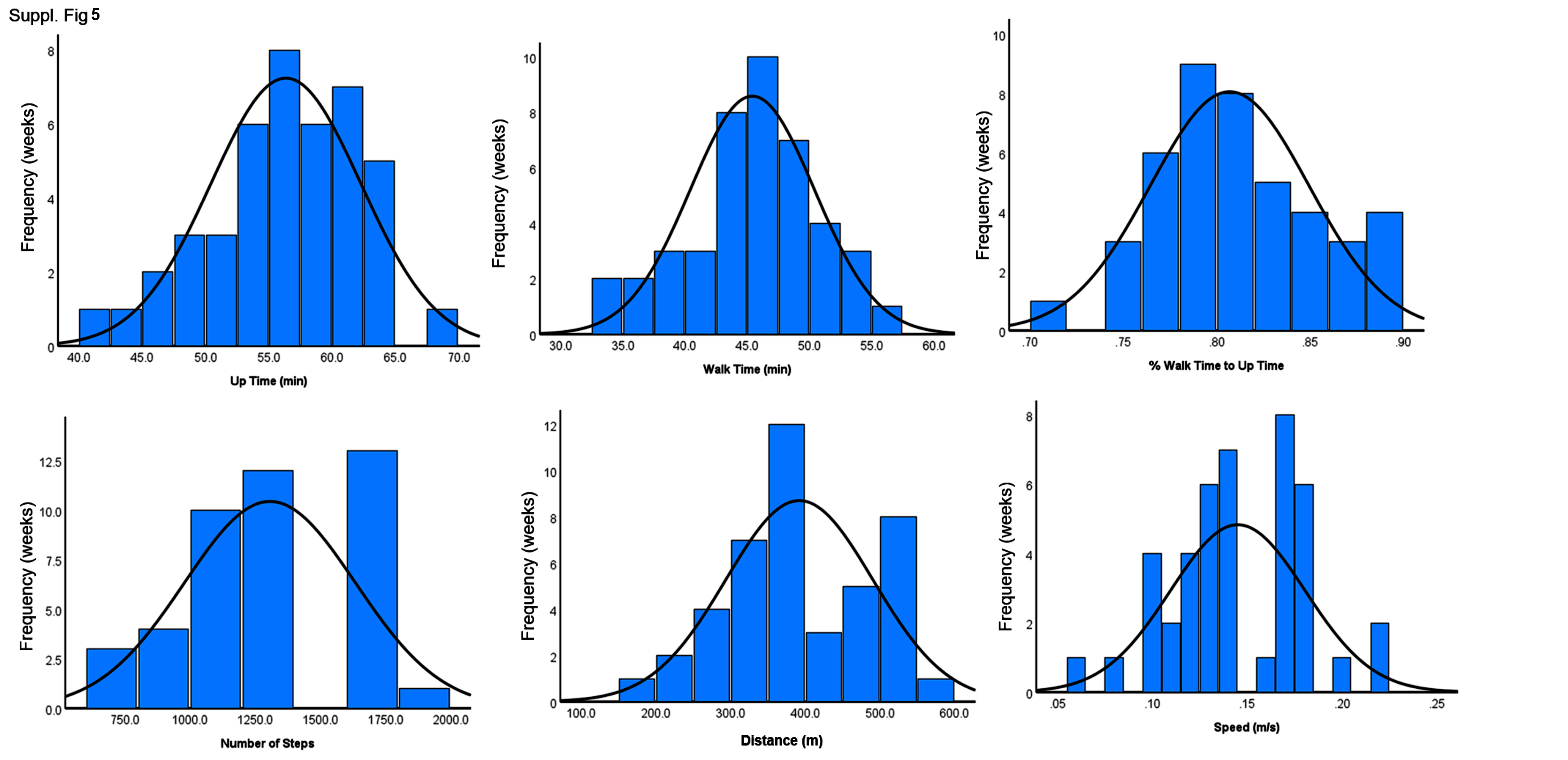

Supplement: Supplementary file 1 [file life-16-00077-s001.zip › 0884M_Supplementary Figure S5.tif]

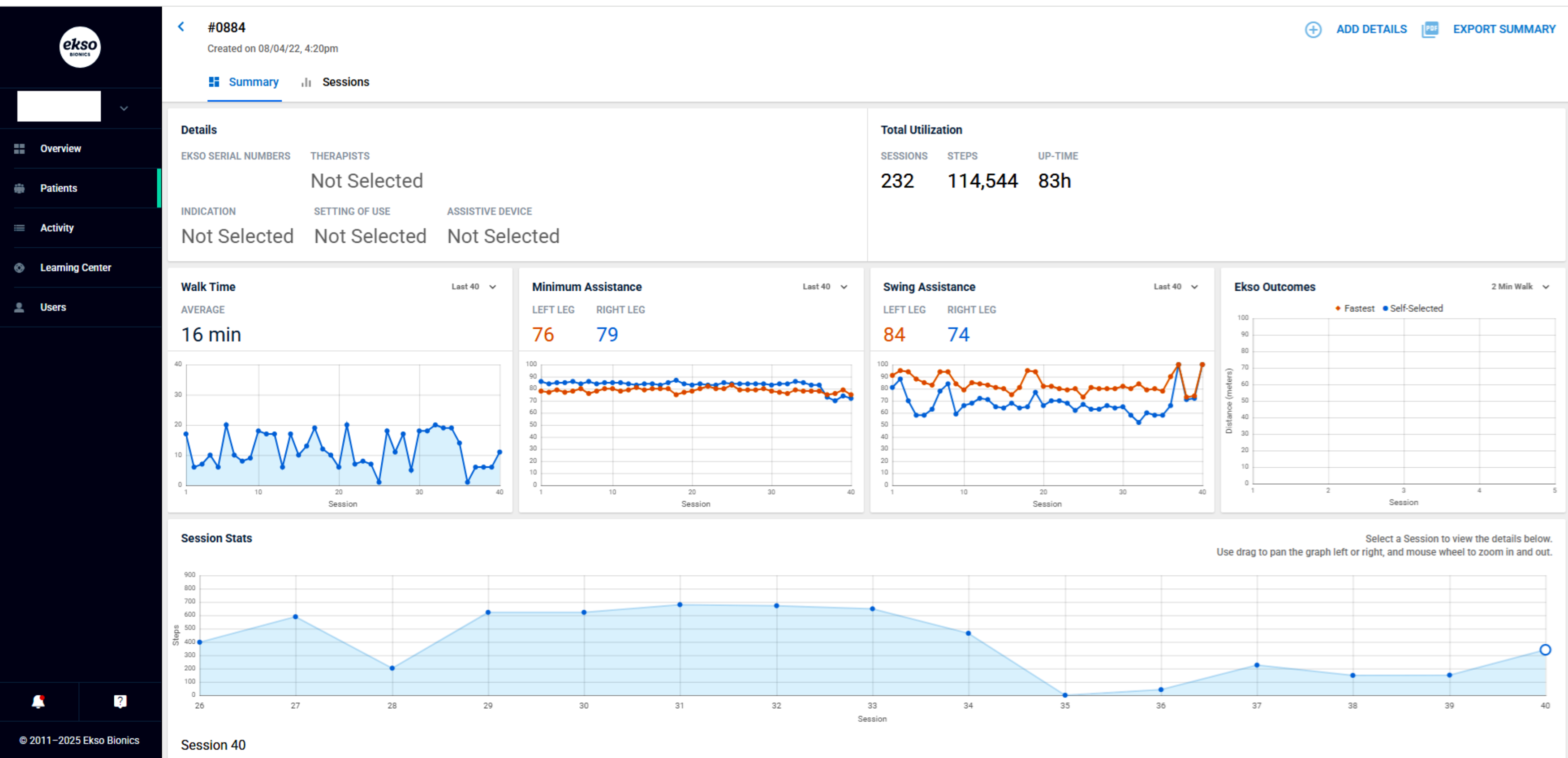

Supplement: Supplementary file 1 [file life-16-00077-s001.zip › Supplementary Figure S1.tif]

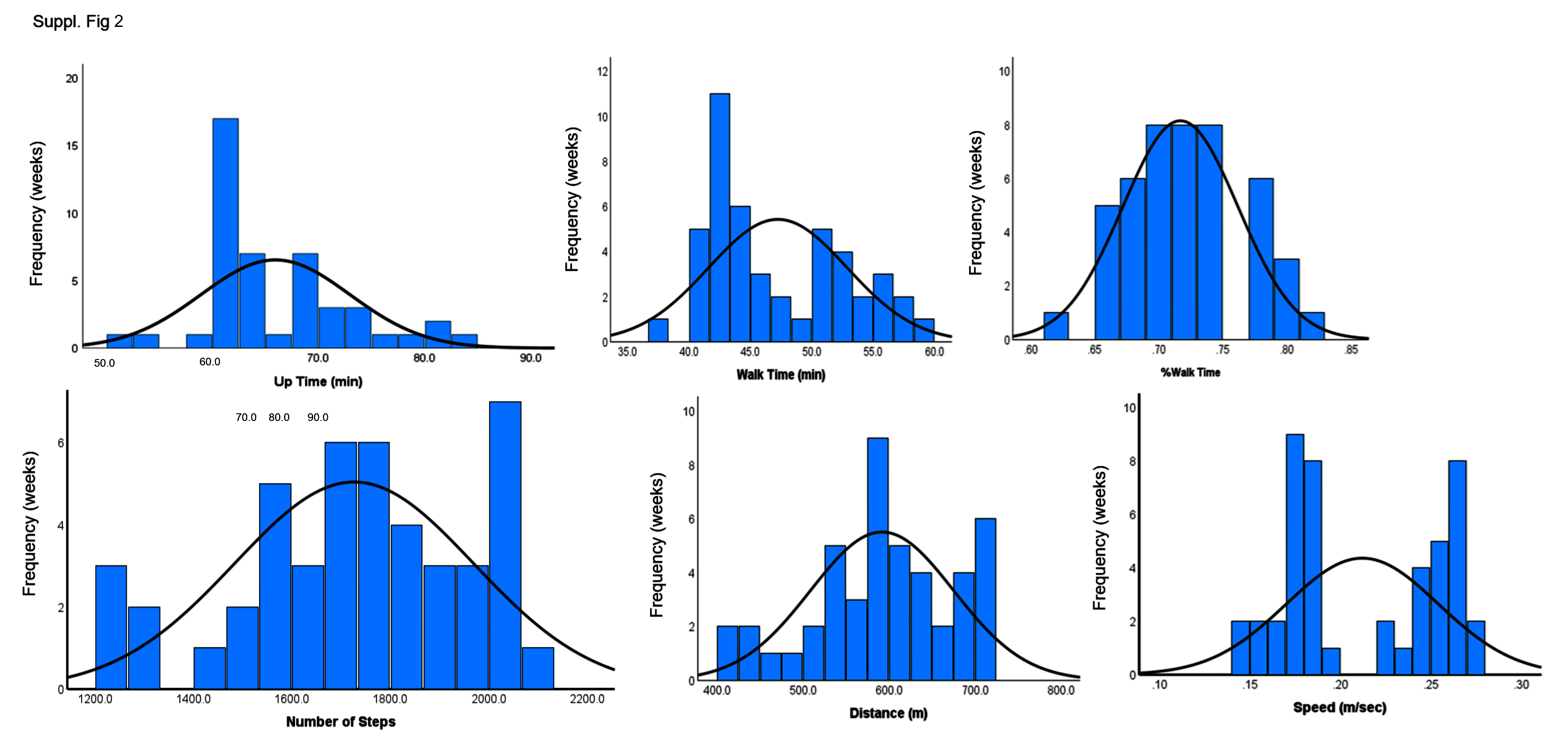

Supplement: Supplementary file 1 [file life-16-00077-s001.zip › Supplementary Figure S2.tif]
